# Terrane accretion explains thin and hot ocean-continent back-arcs

**DOI:** 10.1126/sciadv.adq8444

**Published:** 2025-04-25

**Authors:** Zoltán Erdős, Ritske S. Huismans, Sebastian G. Wolf, Claudio Faccenna

**Affiliations:** ^1^ HUN-REN Institute of Earth Physics and Space Science (HUN-REN EPSS), Sopron, Hungary.; ^2^ Department of Geophysics and Space Science, Eötvös Loránd University, Budapest, Hungary.; ^3^ GFZ Helmholtz Centre for Geosciences, Potsdam, Germany.; ^4^ Department of Earth Sciences, University of Bergen, Bergen, Norway.; ^5^ Department of Science, University Roma Tre, Roma, Italy.

## Abstract

The origin of hot ocean-continent back-arc regions with very thin mantle lithosphere and very high surface heat flow in both extensional and contractional ocean-continent subduction systems is highly enigmatic and unresolved. These first-order characteristics have often been explained with either convective mantle lithosphere removal or by back-arc extension. However, it is unclear what may cause the proposed convective thinning and/or delamination of eclogitic lower crust over very wide regions, whereas back-arc extension is either not observed or insufficient to explain the observed very thin mantle lithosphere. Notably, many of these ocean-continent systems have a long history of terrane accretion. Here, we show, using thermomechanical model experiments, that terrane accretion provides a consistent explanation for the observed key characteristics and naturally leads to rheologically weak back-arcs with continental crust directly on top of the hot sublithospheric mantle.

## Teaser

Accretion of continental terranes during subduction explains hot and thin ocean-continent back-arc systems.

## INTRODUCTION

**Fig. 1.**
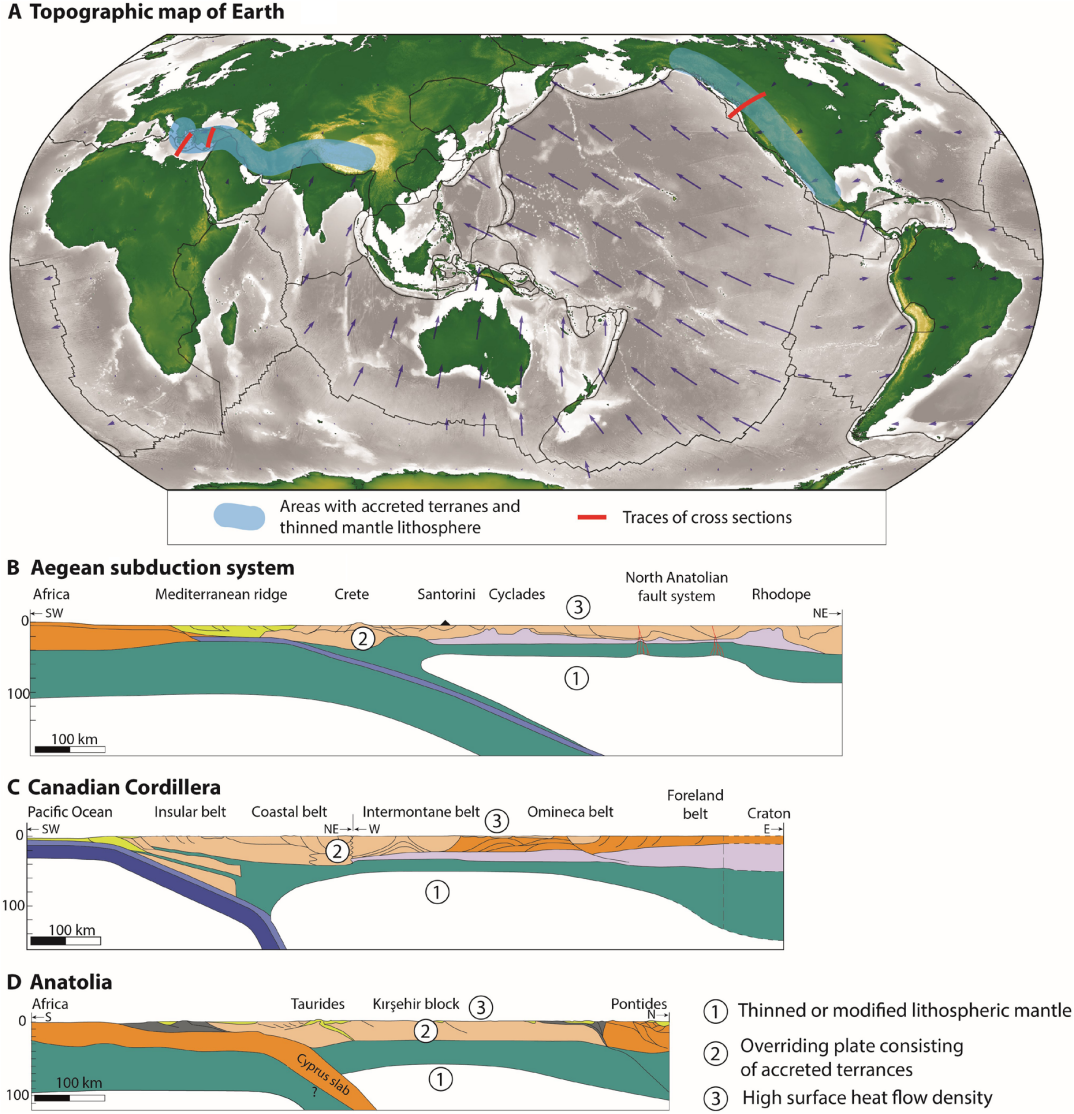
Natural systems with accretionary back-arcs. (**A**) Topographic map of Earth with blue shaded areas highlighting zones with accreted terranes and thinned mantle lithosphere and red lines indicating the locations of the cross sections. Topography and bathymetry are from ETOPO1 (*102*), arrows show relative plate motions in a stable Eurasia reference frame from (*103*), and plate boundaries are from (*104*). (**B**) Lithospheric scale cross section through the Aegean subduction system. The section has been modified after (*8*). The shallow LAB depth under the back-arc has been drawn after (*6*, *32*, *33*). (**C**) Lithospheric scale cross section through the Canadian Cordillera. The section has been redrawn and modified after (*25*). The cratonic part has been added based on (*105*). (**D**) Lithospheric scale cross section through Anatolia. The section was modified after (*15*). The colors correspond to the color scheme used in the model experiments (see Fig. 5: Experimental setup).

Continental back-arcs in the overlying plate of ocean-content subduction systems are common geological features found on Earth (*1*). They exhibit fundamentally different tectonic behaviors ranging from extension (e.g., Aegean Sea), through relative stability (e.g., Canadian Cordillera), to large-scale shortening (e.g., Himalayas). Furthermore, a dynamic deformation history, with alternating phases of shortening, quiescence, and extension is also characteristic (Fig. 1) (*2*, *3*).

In particular, the Aegean Sea is a hot (*4*, *5*), thin (*6*) extensional back-arc system formed in the collision zone between Africa and Eurasia, during the closure of the Tethyan ocean that started at the end of the Cretaceous (*7*–*9*). The Tethyan Ocean was divided into the Vardar, Pindos, and Ionian oceanic domains by small continental blocks (termed here as microcontinental terranes) that were embedded in the oceanic lithosphere. These terranes were accreted to the European margin during the continuous subduction of the encompassing oceanic lithosphere, resulting in discrete shortening events that interrupted the otherwise extensional deformation of the back-arc (*7*–*12*).

The lateral continuation of the Hellenic system in the Anatolian microplate exhibits moderately thickened continental crust with very thin mantle lithosphere below (Fig. 1C) (*13*–*16*). Western and Central Anatolia are made up of the Tavşanlı-Kırşehir and the Anatolide-Tauride terranes that were accreted during the late Cretaceous and early Cenozoic and can to first order be correlated with the accreted terranes in the Hellenic system (*17*–*21*). Shortening associated with subduction-accretion of these blocks continued until Oligocene followed by regional back-arc extension from the late Oligocene to the present in particular in Western Anatolia (*19*–*22*). We note diachroneity of the accretion events between the Aegean, West, and Central Anatolia, with earlier closure of oceanic basins in Anatolia compared to the Aegean (*8*, *16*, *19*, *21*).

The Canadian Cordillera is a long-lived ocean-continent subduction system where a sequence of terrane accretion events started in the Middle Jurassic [~170 million years ago (Ma)] and lasted until the Early Eocene (~56 Ma) when a short extensional event was followed by relative tectonic quiescence (*23*–*26*). Between 100 and 40 Ma, the western part of the Cordillera experienced extensive right-lateral strike-slip faulting that accommodated the northward motion of the accreted terranes relative to North America (*24*, *25*). The crustal thickness varies between normal to moderately thick (30 to 40 km) and shows no clear correlation with the accreted terranes, whereas the lithosphere-asthenosphere boundary (LAB) is imaged at ~60 km depth by seismic refraction data (Fig. 1C) (*25*). Regionally high heat flow density data (*27*, *28*) imply a very thin thermal lithosphere consistent with the seismic data, low elastic thickness (*28*), and a rheologically weak lithosphere.

In summary, these back-arcs are characterized by (i) a shallow LAB extending hundreds of kilometers behind the volcanic arc (*6*, *13*, *14*, *25*, *29*–*33*), (ii) anomalously high surface heat flow density (*4*, *5*, *13*, *27*, *34*–*37*), (iii) weak and tectonically active lithosphere (*13*, *20*, *25*, *26*, *28*, *38*, *39*), and (iv) recurring phases of shortening and extension (*7*, *8*, *20*, *26*). Convective removal of mantle lithosphere (*40*–*44*), delamination of eclogitized lower crust and mantle (*45*–*47*), and back-arc extension (*48*) are the three main mechanisms invoked to explain these characteristics. Numerical modeling has shown that convective removal of mantle lithosphere requires pervasively weakened and compositionally dense mantle lithosphere [e.g., (*41*, *43*)]. It is not clear what may cause substantial weakening and high densities up to 800 km from the trench. Furthermore, the depleted nature of continental mantle lithosphere makes it positively buoyant, with a low propensity for convective destabilization that would require pervasive refertilization. Second, back-arc extension cannot explain almost complete removal of mantle lithosphere beneath moderately thinned continental crust on top. The very thin to absent lithospheric mantle would require a much thinner crust for back-arc extension to be a viable mechanism. Last, delamination of eclogitized lower crust and associated refertilized mantle lithosphere has been suggested as an explanation for absent mantle lithosphere in orogenic back-arc systems such as the Andes [e.g., (*49*, *50*)]. This process may be viable locally behind the arc but requires fluids and the right pressure-temperature conditions associated with crustal thickening. We would not expect these conditions to be present below a vast area of the overriding plate lithosphere far from the trench (*49*). All the abovementioned areas are characterized by a long history of multiple terrane accretion events (*8*, *11*, *19*, *20*, *23*–*25*). However, the potential role of accretion tectonics in shaping the lithospheric architecture of the overriding plate remains enigmatic (*12*, *51*).

Microcontinental terranes are generally made up of buoyant crustal material of varying extent and thickness, embedded within an oceanic plate and accrete to the overlying plate upon collision (*7*, *12*, *51*–*54*). Here, we use two-dimensional (2D) thermomechanical models to investigate the role of continental terrane accretion during ocean-continent subduction. The numerical code solves the momentum, mass, and thermal energy conservation equations in two dimensions (*43*, *55*, *56*). Materials follow frictional-plastic and nonlinear, thermally activated viscous flow laws. In our experiments the computational model domain is 1400 km deep and 3000 km wide. The initial layered geometry consists of a constant viscosity lower mantle overlain by an adiabatic sublithospheric mantle. On the right side of the model, a 120-km–thick idealized continental plate overlies the sublithospheric mantle, whereas on the left side, an 80-km–thick oceanic plate is driven toward the right at a constant velocity. The right-hand boundary is kept fixed, whereas the arbitrary Lagrangian-Eulerian design allows for the free evolution of surface topography. Within the oceanic plate, three 250-km-wide and 30-km-thick microcontinental terranes are embedded allowing for self-consistent modeling of accretion tectonics (see the Materials and Methods on model parameters and detailed description of model setup).

## RESULTS

**Fig. 2.**
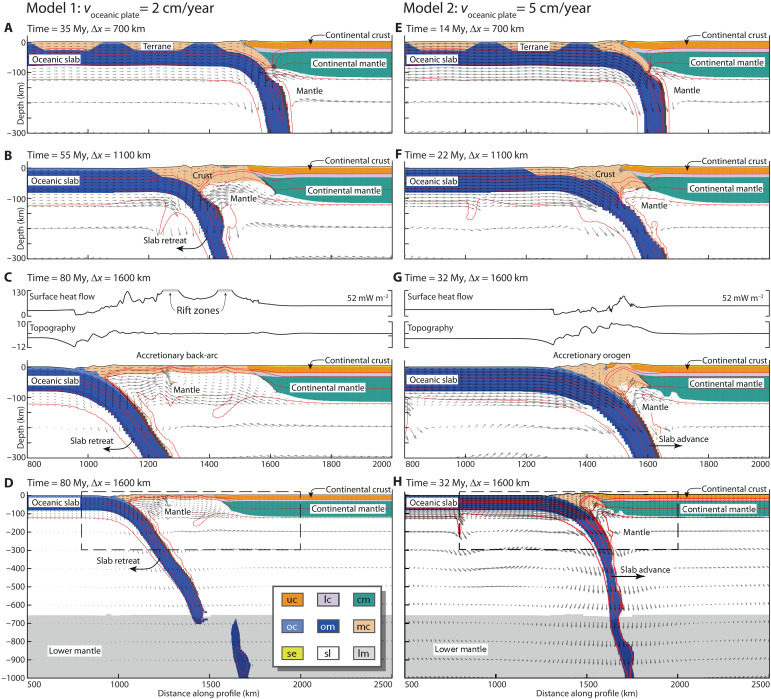
Key stages for Models 1 and 2. One model per column [Model 1 (**A** to **D**) and Model 2 (**E** to **H**)]. The model snapshots display key stages of the models showing material distribution at the same amount of convergence but different times, due to the different convergence velocities (uc, continental upper crust; lc, continental lower crust; cm, continental lithospheric mantle; oc, oceanic crust; om, oceanic lithospheric mantle; mc, microcontinental crust; se, sediment; sl, sublithospheric mantle; lm, lower mantle). The snapshots also include selected isotherms (red lines: 420°, 550°, 900°, and 1330°C) and advection velocities (gray arrows; lengths scale with velocities). Subfigures (A) to (C) and (E) to (G) show a zoomed-in view of the central portion of the models, whereas (D) and (H) show the full model domains at the same stage as (C) and (G). Subfigures (C) and (G) also show surface heat flow density and topography profiles along the zoomed-in section. The vertical axis of the surface heat flow density plots is truncated at 130 mW m^−2^, but in the case of Model 1, the calculated values are locally higher, in the vicinity of two back-arc rifts.

We present first two models that only differ in the velocity of the subducting plate. Model 1 is characterized by a low velocity of the subducting oceanic plate (*v*_*oc*_ = 2 cm year^−1^; Fig. 2, A to D, and movie S1), whereas Model 2 is characterized by a high velocity of the subducting oceanic plate (*v*_*oc*_ = 5 cm year^−1^; Fig. 2, E to H, and movie S2).

Model 1 exhibits four characteristic stages. During Stage 1, the oceanic lithosphere subducts in a stable manner below the overlying continental lithosphere (see movie S1). Stage 2 involves consecutive accretion of the three microcontinents (Fig. 2A). During each accretion event, the continental crust detaches from the underlying mantle lithosphere and is coupled to the overlying plate while the mantle lithosphere continues to subduct into the underlying sublithospheric mantle. During each accretion event the terrane exhibits initial shortening followed by extension associated with rollback of the subducting plate to the seaward edge of the microcontinent. This event is repeated for microcontinents 2 and 3, leading to a broad zone of thick accreted continental crust directly overlying hot sublithospheric mantle. This model displays slab breakoff in the lower part of the upper mantle during microcontinent accretion. During Stage 3, the back-arc region exhibits relative quiescence and stable subduction as the tip of the slab interacts with the 660-km phase discontinuity (Fig. 2B). Stage 4 involves subduction rollback and wide, distributed extension of the back-arc (Fig. 2, C and D). The final configuration shows continuous subduction of oceanic lithosphere from the surface until the transition zone, fragments of the oceanic lithosphere in the lower mantle resulting from slab breakoff and an ~600-km wide back-arc with continental crust overlying hot mantle (Fig. 2, C and D).

Model 2 that has a higher subduction plate velocity of 5 cm year^−1^ exhibits a similar evolution during the initial stages as Model 1 with stable subduction (Stage 1) and accretion of the three microcontinents (Stage 2), with very minor retreat of the subducting slab during each accretion event (Fig. 2, E and F). Following complete accretion of the three microcontinents by 33 million years (Myr), the subducting oceanic plate exhibits very minor retreat resulting in a 300-km-wide region of thick accreted crust underlain by hot mantle and an oceanic slab that is continuous from the surface into the lower mantle (e.g., Stage 3). The final stage is characterized by episodes of alternating extension, quiescence, and shortening (Fig. 2, G and H).

**Fig. 3.**
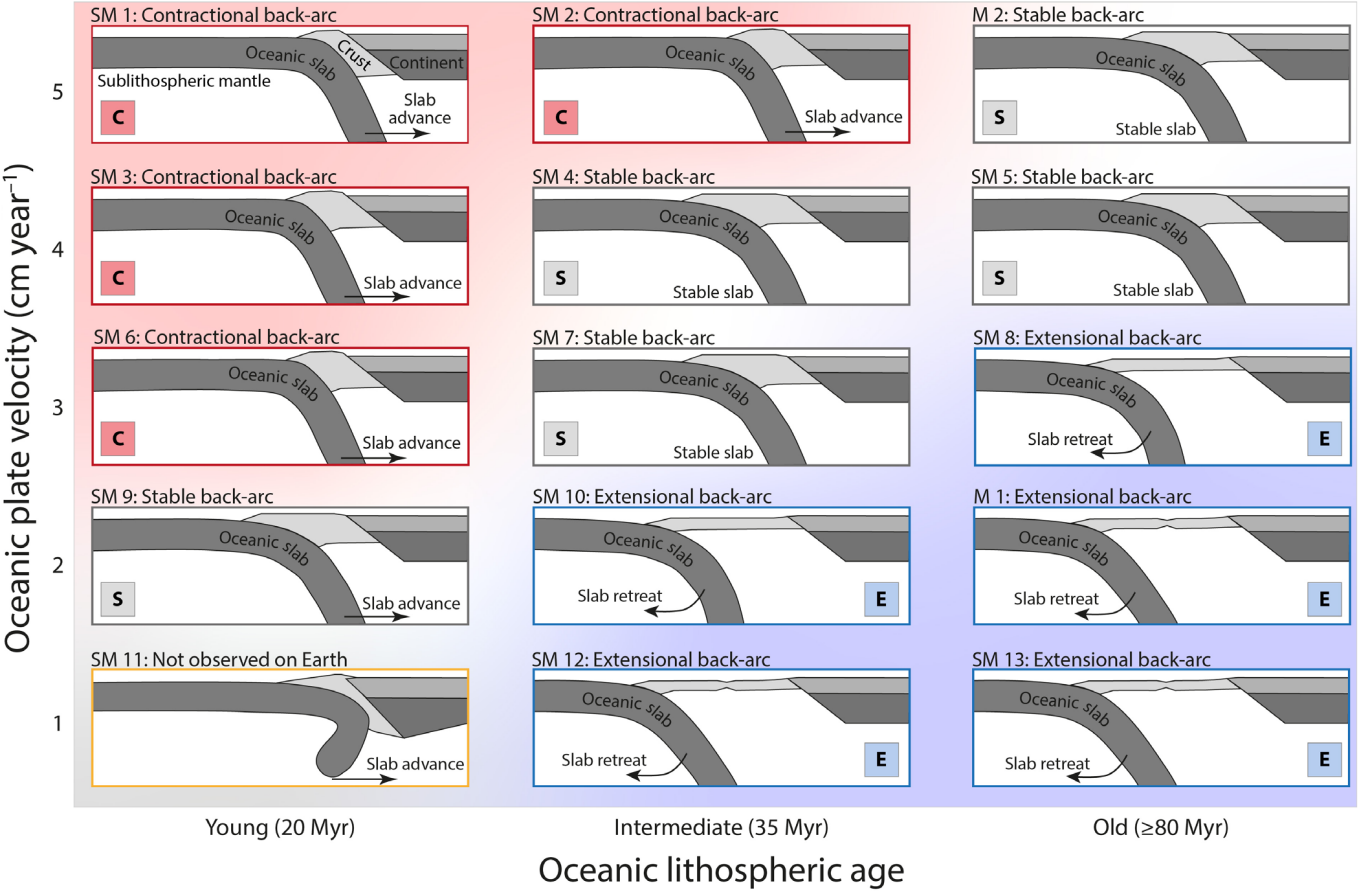
Domain map showing the dominant back-arc behavior of an extended set of models. We varied the velocity of the subducting oceanic plate (vertical axis) and the age of the oceanic lithosphere through the degree of depletion of the oceanic mantle lithosphere (horizontal axis). The values of the degree of depletion representing young (20 Myr), intermediate (35 Myr), and old (≥80 Myr) oceanic lithospheres are 40, 30, and 15 kg m^−3^ respectively. Red background shading and edge color corresponds to back-arc compression/shortening (also denoted with the letter C), whereas blue background shading and edge color corresponds to back-arc tension/extension (also denoted with the letter E). White background shading and black edge color correspond to stable back-arc regime (also denoted with the letter S), whereas model SM11 in the bottom left corner, marked with a yellow edge color, exhibits a behavior that may not be observed on Earth.

Supplementary models SM1 to SM13 test sensitivity to subducting plate velocity (*v*_*oc*_ = 1, 2, 3, 4, and 5 cm year^−1^) and to varying thermal ages for the oceanic lithosphere (80, 35, and 20 Myr) represented here by variable degrees of depletion of the subducting oceanic mantle lithosphere (Δρ = 15, 30, and 40 kg m^−3^). This is a simple approach that does not take into account oceanic lithospheric thickness changes or the dependence of depletion on the mantle potential temperature [e.g., (*57*)] but captures the first-order effect of plate-age related buoyancy variations. These models show that the overlying plate exhibits back-arc extension for moderate to low subducting plate velocities, whereas higher velocities are associated with back-arc stability or shortening. The thermal age of oceanic lithosphere offsets the transition of the mode of back-arc deformation, with models characterized by younger oceanic lithosphere resulting in back-arc shortening for lower subduction velocities (see Fig. 3, table S1, and movies S3 to S15 for the parameters varied between each model of the parameter study).

## DISCUSSION

### Summary of model results

The model results show that accretion of multiple microcontinental terranes can naturally lead to continental back-arc regions where the crust overlies hot mantle (Fig. 2). The tectonic style of deformation in our models may vary from extension, through dynamic stability, to shortening, and is to first order controlled by the subducting oceanic plate velocity relative to the vertical natural sinking velocity of the slab with respect to a stable overriding plate. The natural sinking velocity is controlled by the negative buoyancy of the subducting oceanic slab and the viscosity of the upper and lower mantle and evolves through time. When the subducting oceanic plate velocity is lower than the natural sinking velocity of the slab, the slab-pull force strongly dominates the system resulting in back-arc extension. The resistance associated with microcontinent collision impedes slab retreat and leads to short phases of overlying-plate shortening (see movie S1). In contrast, a subducting oceanic plate velocity higher than the natural sinking velocity prevents slab retreat, leading to stable and/or shortening back-arc regions without a mantle-lithospheric root (see movie S2). The potential for a slab to advance or retreat depends on the ratio between the subducting plate velocity and the natural sinking velocity of the slab as demonstrated in modeling experiments (*58*). In both end-member scenarios, interaction of the oceanic slab with the 660-km phase transition zone, linked with resistance to penetrating the high viscosity lower mantle, induces transient phases of slab advance and retreat associated with secondary shortening and extension in the back-arc region. The buoyancy contrast between oceanic lithosphere and the ambient mantle varies with plate age through plate thickness and through compositional depletion. Consequently, varying the degree of compositional depletion of the oceanic mantle lithosphere affects the sinking velocity and therefore the transition from slab retreat and back-arc extension to slab stability and/or advance and back-arc shortening (Fig. 3). Although we have not explored the role of overriding-plate velocity here, this provides an additional control on the tectonic style of ocean-continent subducting systems, where overriding plate movement toward the trench will inhibit overriding plate extension and promote shortening [e.g., (*43*)]. We note that the accretion of continental terranes and the associated delamination of their mantle-lithospheric root naturally lead to a weak overlying plate, which is a prerequisite for deformation of the back-arc region [e.g., (*43*)].

### Implications for natural systems

The models presented here show that (i) a shallow LAB extending hundreds of kilometers behind the volcanic arc, (ii) anomalously high surface heat flow density, (iii) weak and tectonically active lithosphere, and (iv) recurring phases of shortening and extension are all inherent features of accretion tectonics along ocean-continent subduction zones. The Hellenic region (Fig. 1A) (*7*) provides a type example of an ocean-continent subduction system characterized by an overall low subducting plate velocity and slab retreat. At present day, it exhibits thin extended crust overlying hot upper mantle and a continuous subducting slab beneath the Aegean region reaching the lower mantle (*6*), following accretion of two continental terranes during the Tertiary (*22*, *59*–*63*). It has been suggested that the single, continuous subducting slab implies efficient delamination and decoupling between the accreted crustal terranes and the subducting mantle (*7*, *12*). Geological data suggest that both accretion events were followed by slab rollback, back-arc extension, high-temperature metamorphic core-complex formation, and contemporaneous emplacement of hot asthenosphere close to the base of the crust (*7*). The final phase characterized by slab rollback starting at around 30 to 35 Ma coincides with a decrease in convergence velocity between Africa end Eurasia from 2 to less than 1 cm year^−1^ (*7*, *64*). Such a change in the velocity of the oceanic plate in our models would lead to slab rollback as inferred for this natural system. Model 1 reproduces the first-order characteristics of this area including (i) shortening and mountain building prior the trench-retreat and extension, (ii) present day extended thin crust and absence of mantle lithosphere, (iii) high surface heat flow in the back-arc, and (iv) the presence of oceanic material along the interfaces of the accreted terranes (*6*, *8*, *9*, *64*–*67*).

The lateral continuation of the Hellenic system in the Anatolian plateau exhibits moderately thickened continental crust with very thin mantle lithosphere below (Fig. 1C). The Anatolian microplate is made up of Eocene and younger orogenic belts that were formed during a series of accretion events of Gondwana-derived ribbon continents, similar to the Aegean area (*17*–*19*, *68*). The two microcontinental terranes in Western and Central Anatolia can be correlated with those in the Aegean domain, but their accretion is diachronous with earlier collision in Anatolia compared to the Aegean (*21*). A range of observations including seismic Pn velocity and Sn attenuation tomography (*69*, *70*) and middle Miocene to recent alkaline volcanism in Central Anatolia (*71*) show that the uppermost mantle beneath much of Central and Western Anatolia is anomalously hot and thin (*19*). Previous studies suggest that delamination of the subcontinental lithospheric mantle beneath the orogenic belt may explain these observations (*19*, *71*). The model results presented here show that delamination of the subducting mantle lithosphere is a natural consequence following terrane accretion that results in moderately thick continental crust with very thin mantle lithosphere below (e.g., Fig. 2B), providing a consistent mechanism for the first-order characteristics of this system. In Eastern Anatolia, geological reconstructions suggest a more complex evolution involving potentially multiple subduction zones. Although this area is similarly characterized by very thin mantle lithosphere, the role of accretion tectonics is less obvious (*16*, *19*, *20*).

The Canadian Cordillera, in contrast, is a type example of an ocean-continent subduction system characterized by a high subducting plate velocity and motion of the overriding plate toward the trench, with moderately thickened continental crust, very thin to absent mantle lithosphere below (*25*), and a long history of terrane accretion (*38*, *72*, *73*). Major accretion events in this area occurred between the middle Jurassic and the Paleocene (170 to 60 Ma). These were followed by a complex tectonic history involving strike slip deformation and a phase of large-scale extension and crustal thinning during the Early Eocene (around 56 Ma). Subsequently, this area is characterized by relative quiescence and continuing stable subduction along the western margin (*23*–*25*). Model 2 with a high subducting plate velocity reproduces the first-order characteristics of this area (Fig. 1B) including (i) relative stable subduction following terrane accretion, (ii) a region of thick back-arc crust on top of the hot mantle, and (iii) episodic extension and shortening events affecting the overlying plate.

Anatolia and the Canadian Cordillera both experienced phases of large-scale transcurrent deformation that are not captured by the 2D modeling approach used here (*17*, *24*, *25*, *68*). For instance, in the case of Anatolia, the westward extrusion of the domain toward the Aegean initiated at ~13 Ma, much after the main accretion events, and is still ongoing, together with active shortening in Eastern Anatolia (*20*). In the case of the Canadian Cordillera, the northward translation of the accreted terranes occurred after the conclusion of the major accretion events, between 100 and 40 Ma (*25*, *26*). These translational tectonic aspects of the two systems are conservative processes and cannot explain accretion, crustal thickening or thinning, which result from trench-orthogonal deformation. In addition, very thin to absent mantle lithosphere in these systems cannot be explained by transcurrent deformation. A 2D model approach is consequently suitable to address the first-order trench-orthogonal aspects of these systems.

Many accretionary orogens are characterized by low-pressure, high-temperature granulite metamorphic assemblages. The metamorphic conditions indicated by these granulite facies rocks are commonly synchronous with structural evidence for crustal thickening but were considered too hot to have been formed during continental collision (*3*). This apparent contradiction is known as the granulite conundrum (*2*, *3*) and has been explained with repeated alternation (tectonic switching) between lithospheric extension and contraction. Tectonic switching involves short episodes of shortening interspersed with stretching phases that allow heating of thickened orogenic crust. Subduction and accretion of oceanic plateaus and associated flat-slab subduction were invoked as an explanation for the shortening episodes and slab retreat for the extensional phases (*3*, *74*). Recent modeling studies suggest that delamination of continental mantle lithosphere during the formation of Archean and Precambrian ultrahot orogens explains granulite metamorphism in these settings (*75*, *76*). However, these observations are also consistent with delamination and subduction of the mantle lithosphere during microcontinent accretion as shown in our models, which provides an explanation for simultaneous shortening and heating in these settings and the high temperatures needed to form granulite-facies rocks. We note that the accretion of terranes in our models is associated with short-lived episodes of extension alternating with shortening. However, the extensional episodes provide only a secondary control on mantle heating.

**Fig. 4.**
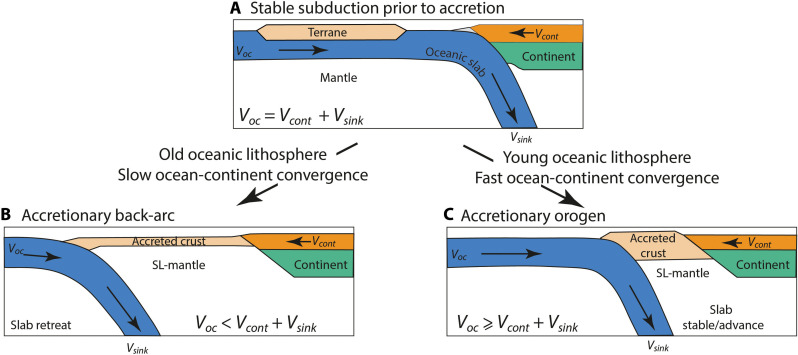
Contrasting styles of back-arcs. Properties and conditions for two types of back-arcs following terrane accretion. (**A**) Stable subduction prior to terrane accretion. (**B**) Low ocean-continent convergence velocity and/or subduction of old dense oceanic lithosphere promote slab roll back following terrane accretion and formation of an accretionary back-arc. (**C**) High ocean-continent convergence velocity and/or subduction of young oceanic lithosphere promote stable subduction or slab advance following terrane accretion and formation of an accretionary orogen. *V_oc_* denotes oceanic plate velocity, *V_cont_* denotes overriding continental plate velocity (note that *V_cont_* has an opposite sign to *V_oc_*), and *V_sink_* denotes the natural sinking velocity of the subducting slab.

In summary, continental terrane accretion characterized by a low ocean-continent convergence velocity is associated with subduction roll-back and promotes formation of accretionary back-arcs with wide zones of thin, extended continental crust, and an anomalously shallow LAB below (Fig. 4B). In contrast, continental terrane accretion characterized by a high ocean-continent convergence velocity leads to stable subduction or trench advance and promotes formation of accretionary orogens with thick crust directly on top of the hot sublithospheric mantle (Fig. 4C). In such systems, the delicate balance between subducting plate and overriding plate velocity and the natural sinking velocity of the slab provides a dynamic equilibrium that allows intermittent periods of extension, stability, and shortening, as observed for example in the Hellenic region (*8*). We speculate and predict that the highly dynamic deformation style shown here can also be observed in other ocean-continent and continent-continent collision systems with accreted terranes such as in the Zagros mountain belt after the accretion of the Cimmerian microcontinent (*77*, *78*) or in Tibet that is characterized by the amalgamation of multiple accreted terranes in an overall convergent setting (*79*, *80*). In particular, the Tibet-Himalayan system has a very long history of terrane accretion prior to India-Eurasia collision and exhibits very thin lithospheric mantle beneath parts of the Tibetan plateau (*80*, *81*). It also shows underthrusting of the Indian continental lithosphere below the thickened, accreted terranes forming the southern part of the orogen. Our models provide a plausible explanation for the absence of mantle lithosphere below the accretionary orogen that may also have facilitated the underthrusting of the depleted, buoyant Indian mantle lithosphere. However, the models presented here are not aimed to reproduce the complex deformation history of Tibet-Himalayan system, which is beyond the scope of this paper and provides a promising direction for future work.

The models presented here provide a simplified representation of ocean-continent subduction systems and accretion tectonics. Their main limitation is that they are 2D, whereas some aspects of subduction and terrane accretion dynamics are more appropriately explored in 3D. They are most applicable to wide subduction zones, where toroidal mantle flow has a limited effect (*82*, *83*). The 2D nature of the models implies that the along-strike width of the microcontinents is comparable to the width of the subduction zone (*82*, *83*). A narrow microcontinent embedded in a wide subducting ocean will only interact with a limited segment of the subduction zone, creating complex 3D effects with transfer zones along the lateral boundaries of the terrane (*84*). However, in all three natural systems discussed here, the width of the subduction zone and the width of the accreting terranes is comparable (*8*, *19*, *28*), and in such cases, our results are applicable. Last, we acknowledge, that decoupling of the mantle lithosphere from the accreted terranes might be inhibited in cases (i) where it is highly depleted and buoyant, leading to underthrusting and flat-slab subduction, or (ii) when the terrane crust is strongly coupled to its mantle-lithospheric root, leading to terrane subduction (*52*, *85*). The former mechanism has been inferred for both the southern part of the Tibet-Himalayan orogen, and the Laramide orogen and has been demonstrated as a viable mechanism by recent numerical modeling studies (*81*, *86*).

The models presented here demonstrate that accretion of microcontinents along ocean-continent subduction systems provides a self-consistent explanation for enigmatic observations from thin and hot ocean-continent back-arc regions. These observations include (ii) the absence of mantle lithosphere extending hundreds of kilometers behind the arc, (ii) anomalously high surface heat flow densities, (iii) a weak and tectonically very active and dynamic back-arc environment, and (iv) recurring phases of shortening and extension. These first-order characteristics are a direct consequence of terrane-accretion, do not require large-scale back-arc extension or convective removal of a weak mantle-lithospheric root, and can remain relatively stable for millions of years after the accretion events. The results presented here provide a predictive framework for understanding the long-term dynamics of ocean-continent subduction systems and offer a coherent explanation for their key characteristics.

## MATERIALS AND METHODS

### Experimental design

We use the 2D arbitrary Lagrangian-Eulerian, finite-element model FANTOM (*42*, *43*, *55*, *56*, *87*), computing thermomechanically coupled, incompressible, plane strain, viscous-plastic creeping flows to investigate microcontinental terrane accretion during ocean-continent subduction. FANTOM solves the Navier-Stokes equations coupled to the heat transport equation in 2D, using the plane strain approximation where the mechanical and thermal systems are coupled through temperature-dependent rheologies and densities and are solved sequentially for each time step.

When stress is below yield, deformation occurs by viscous flow and follows temperature-dependent nonlinear power law rheologies. The effective viscosity η_eff_ is specified as

$$\eta_{\text{eff}}=f\cdot A^{-1/n}\cdot {{\overset{\cdot }{\varepsilon }}_{\mathrm{II}}}^{\left(1-n\right)/2n}\cdot \text{exp}\left(\frac{Q+\mathrm{Vp}}{\mathrm{nRT}}\right)$$ (1)

where *A* is the pre-exponential scaling factor, *n* is the power law exponent, ${\overset{\cdot }{\varepsilon }}_{\mathrm{II}}$ is the second invariant of the deviatoric strain rate tensor, *Q* is the activation energy, *V* is the activation volume, *p* is the pressure, *T* is the temperature, and *R* is the universal gas constant. The material-specific values for the parameters *A*, *n*, *Q*, and *V* are based on laboratory measurements and are provided in Table 1. The effective viscosity of quartz-dominated rocks is characterized by large uncertainties due to compositional differences (*88*), whereas laboratory data show that water-saturated olivine is 5 to 20 times weaker than dehydrated olivine at the same strain rate (*89*, *90*). Furthermore, the diabase used in the laboratory experiments was drier, and consequently stronger, than the typical lower crust (*40*). To effectively account for such variability, we use a pre-exponential scaling factor (*f*) to adjust individual flow laws.

**Table 1.** Material properties. Notes: WQ, Wet Quartz flow law (*97*); DMD, Dry Maryland Diabase flow law (*96*); WO: Wet Olivine flow law (*90*).

	Thickness	Reference density	Friction angle	Cohesion	Flow law	Scaling factor	*A*	*Q*	*n*	*V*	Viscosity range	Heat capacity	Heat conductivity	Thermal expansion	Heat productivity
Unit	km	kg m^−3^	degree	Pa	–	–	Pa^−*n*^ s^−1^	J mol^−1^	–	m^3^ mol^−1^	Pa s	m^2^ K^−1^ s^−2^	W m^−1^ K^−1^	K^−1^	W m^−3^
Continental plate															
Sedimentary cover	4	2800	8°–2°	2 × 10^7^–4 × 10^6^	WQ^1^	1	8.57 × 10^−28^	2.23 × 10^5^	4	0	10^19^–10^27^	750	2.25	3 × 10^−5^	1.1 × 10^−6^
Upper crust microcontinent	24	2800	15°–2°	2 × 10^7^	WQ^1^	1	8.57 × 10^−28^	2.23 × 10^5^	4	0		750	2.25	3 × 10^−5^	1.1 × 10^−6^
Lower crust	12	3000	15°–2°	2 × 10^7^	DMD^2^	0.1	5.78 × 10^−27^	4.85 × 10^5^	4.7	0		750	2.25	3 × 10^−5^	5 × 10^−7^
Mantle lithosphere	84	3380 (3360)^†^	15°–2°	2 × 10^7^	WO^3^	5	1.76 × 10^−14^	4.3 × 10^5^	3	1.1 × 10^−5^		1250	2.25	3 × 10^−5^–4 × 10^−5^	0
Oceanic plate															
Crust	9	2900	7°–1°	2 × 10^7^	DMD^2^	0.1	5.78 × 10^−27^	4.85 × 10^5^	4.7	0	10^19^–10^27^	750	2.25	3 × 10^−5^	0
Mantle lithosphere	71	3380 (3365)^‡^	15°–2°	2 × 10^7^	WO^3^	5	1.76 × 10^−14^	4.3 × 10^5^	3	1.1 × 10^−5^		1250	2.25	3 × 10^−5^–4 × 10^−5^	0
Eclogite from sediment	–	3365	15°–2°	2 × 10^7^–4 × 10^6^	WQ^1^	0.5	8.57 × 10^−28^	2.23 × 10^5^	4	0		750	2.25	3 × 10^−5^	0
Eclogite from crust	–	3380	15°–2°	2 × 10^7^	DMD^2^	0.1	5.78 × 10^−27^	4.85 × 10^5^	4.7	0		750	2.25	3 × 10^−5^	0
Asthenosphere															
Sublithospheric mantle	536 (580)^*^	3380	15°–2°	2 × 10^7^	WO^3^	1	1.76 × 10^−14^	4.3 × 10^5^	3	1.1 × 10^−5^	10^19^–10^27^	1250	2.25–52.0	3 × 10^−5^–4 × 10^−5^	0
Constant viscosity lower mantle	740	3630	–	–	–	–	–	–	–	–	2 × 10^21^	1250	2.25–52.0	3 × 10^−5^–4 × 10^−5^	0
Slab in the lower mantle	–	3630	15°–2°	2 × 10^7^	WO^3^	5	1.76 × 10^−14^	4.3 × 10^5^	3	1.1 × 10^−5^	10^19^–10^27^	1250	2.25	3 × 10^−5^–4 × 10^−5^	0

*Value in parenthesis shows the thickness of the sublithospheric mantle under the undeformed oceanic lithosphere.

†Value in parenthesis shows the density of the depleted layer of continental mantle lithosphere.

‡Value in parenthesis shows the density of the depleted layer of oceanic mantle lithosphere.

**Fig. 5.**
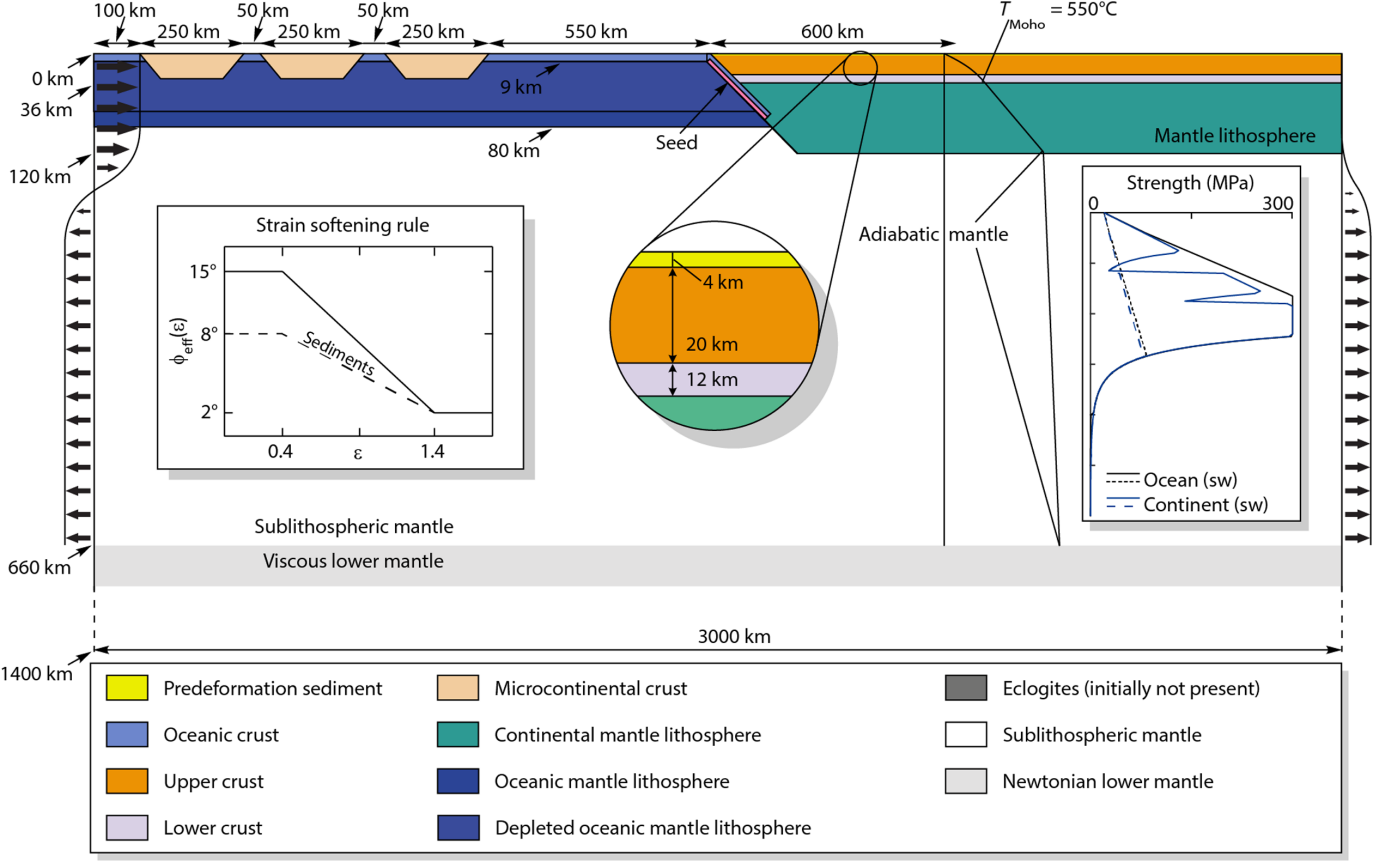
Experimental setup showing the initial geometry and the velocity boundary conditions used in all presented models. The figure also presents the applied strain-softening rule, the initial continental temperature gradient with depth, and the continental and oceanic lithospheric strength profiles calculated analytically, using a constant strain rate value of 10^−15^ s^−1^.

When stress is above yield, deformation occurs by frictional-plastic yielding. This is modeled with a pressure-dependent Drucker-Prager yield criterion, equivalent to the Coulomb yield criterion in two dimensions. Yielding occurs when

$${\left(J_{2}^{\prime }\right)}^{1/2}=p\;\text{sin}\phi_{\text{eff}}+C\text{cos}\phi_{\text{eff}}$$ (2)

where $J_{2}^{\prime }$ is the second invariant of the deviatoric stress, $\phi_{\text{eff}}$ is the effective internal angle of friction (that includes the effect of pore-fluid pressure), and *C* is the cohesion. With appropriate choice of *C* and $\phi_{\text{eff}}$, this yield criterion approximates the effect of pore-fluid pressure and frictional sliding in rocks. Mechanisms such as fluid-pressure variations (*91*), grain-size reduction, and mineral transformations (*92*) may reduce the frictional-plastic strength. The effect of these plastic strain-softening mechanisms is introduced into the model through a linear decrease in the internal angle of friction from 15° to 2° and—for the sedimentary layers—a simultaneous decrease in cohesion from 20 to 4 MPa as the second invariant of deviatoric strain varies from 0.05 to 1.05 (see Fig. 5). We note that $\phi_{\text{eff}}\left(\varepsilon \right)=15{}^{\circ}$ corresponds to the effective friction angle when the pore-fluid pressure is approximately hydrostatic.

In addition to solving the Navier-Stokes equations for viscous-plastic flows, the heat transport equation is solved in two dimensions (*i* = 1, 2)

$$\rho \cdot C_{p}\left(\frac{\partial T}{\partial t}+v_{i}\cdot \frac{\partial T}{\partial x_{i}}\right)=\frac{\partial k}{\partial x_{i}}\frac{\partial T}{\partial x_{i}}+H+v_{z}\alpha \rho \mathrm{gT}$$ (3)

where ρ is the density, *C_p_* is the heat capacity, *t* is the time, *v_i_* and *x_i_* are the velocity and spatial components in the *i* direction, *k* is the thermal conductivity, and *H* is the heat production per unit volume. Thermal conductivity is dependent on temperature in the sublithospheric mantle and constant for all other materials. The last component on the right-hand side describes adiabatic heating and cooling when the material moves vertically at a velocity *v_z_*, where α is the thermal expansion coefficient. The mechanical and thermal systems are coupled through the temperature dependent rheologies and densities; they are solved sequentially for each model time step. The temperature dependence of the densities follows the equation

$$\rho \left(T\right)=\rho_{0}\left[1-\alpha \left(T-T_{0}\right)\right]$$ (4)

where ρ_0_ is the reference density at the reference temperature *T*_0_ = 0°C. For mantle materials, the thermal expansion coefficients are *P*-*T* dependent with a linear increase from 3 × 10^−5^ to 4 × 10^−5^ K^−1^ in the temperature range of 500 to 2000 K and a linear decrease by a factor 1 to 0.5 from 0 to 45 GPa (*93*–*95*). Furthermore, to mimic active mantle convection at high Nusselt number, the thermal conductivity linearly increases from 2.25 to 52.0 W m^−1^ K^−1^ in the temperature range of 1335° to 1345°C specifically for the sublithospheric mantle and the constant viscosity lower mantle.

### Materials and geometry

The experimental setup is presented in Fig. 5. The computational domain is 3000 km wide and 1400 km deep. The initial geometry consists of a constant viscosity lower mantle overlain by an adiabatic sublithospheric mantle that has a rheology of Wet Olivine (*90*). Above these two layers, on the right side of the model lies the overriding continental plate. Its mantle lithosphere is 84 km thick and has a rheology of Wet Olivine with a scaling factor *f* = 5. The crust of this plate consists of a 12-km-thick lower crust that has a Dry Maryland Diabase rheology (*96*) with a scaling factor of *f* = 0.1, a 20-km-thick upper crust that has a Wet Quartz rheology (*97*) with a scaling factor of *f* = 1, and a 4-km-thick sedimentary cover that has the same Wet Quartz rheology as the upper crust but with a lower initial frictional-plastic strength (see Fig. 5).

To the left of the overriding plate lies an oceanic domain. Its mantle lithosphere is 71 km thick (Wet Olivine with a scaling factor *f* = 5) and contains a 60-km-thick upper layer that is depleted. For experiments SM 1, 3, 6, 9 and 11, the degree of depletion is 40 kg m^−3^, whereas for experiments SM 2, 4, 7, 10 and 12, the degree of depletion is 30 kg m^−3^. For the rest of the experiments, including the two in presented in the main text, the degree of depletion is 15 kg m^−3^. Above the oceanic mantle lithosphere lies a 9-km-thick oceanic crust (Dry Maryland Diabase with a scaling factor *f* = 0.1) within which microcontinental terrains are embedded (Wet Quartz with a scaling factor *f* = 1). These terrains are 250 km wide and 30 km thick with 30° dipping margins on both sides. The oceanic basin between the first microcontinent and the overriding plate edge is 500 km wide, whereas the oceanic basins between terrains are 50 km wide.

The boundary between the oceanic and the continental domains is dipping 35° to the right. At this divide, the model is seeded with an inclined, strain-weakened oceanic crustal domain underneath the overriding plate to allow for oceanic subduction initiation. All the specific material properties for each material and the references of the specific flow laws can be found in Table 1.

Upon reaching the temperature-pressure conditions for the basalt-eclogite phase change, the oceanic crust is automatically transformed into eclogite (*98*). Eclogite has the same viscous flow law as the oceanic crust but a different density. All slab and mantle materials are subject to a reversible phase transition at the 660-km discontinuity, corresponding to the breakdown of ringwoodite to bridgemanite and magnesiowüstite (*99*, *100*). For simplicity, all materials that make up the subducting slab are converted to one lower mantle material, which has the viscous flow law of the oceanic lithosphere. The phase changes do not account for latent heat and are not mass conserving, but they are assumed to capture the first-order effects of important metamorphic phase changes (*43*).

The rheology of the lower mantle is subject to substantial uncertainties with viscosity estimates ranging between 1 × 10^21^ and 5 × 10^22^ Pa s (*99*, *101*). Here, we use a uniform viscosity of 3 × 10^21^ Pa s. This choice results in a viscosity increase at the 660-km discontinuity of approximately a factor of 30. This higher viscosity of the lower mantle relative to the sublithospheric upper mantle (see fig. S1 for snapshots of the density and viscosity structures of Model 1) results in more shear resistance and hence lower sinking velocity for the slab upon crossing the phase transition zone. The shear resistance and the pull of the slab in the lower mantle have a similar order of magnitude.

### Numerical resolution

The Eulerian computational grid consists of 1250 cells in the horizontal direction (giving a resolution of 2.4 km) and 257 cells in the vertical direction. The vertical resolution is variable, with 127 elements covering the bottom 1270 km of the model (giving a vertical resolution of 10 km), whereas the remaining 130 elements located in the top 130 km of the model (giving a vertical resolution of 1 km). The grid refinement was used to increase the resolution in the lithosphere but attain a reasonable computation time.

### Boundary conditions

The initial temperature field is calculated analytically and, for the continental lithosphere, increases with depth from the surface (*T*_0_ = 0°C) to the base of the crust (*T*_m_ = 550°C) and to the base of the lithosphere (*T*_lab_ = 1330°C) from where it adiabatically increases to the base of the model domain (*T*_base_ = 1850°C) (see the initial temperature profile in Fig. 5). The initial temperature field of the oceanic domain is very similar, but the lack of heat-production in the oceanic crust results in a linear geothermal gradient. The bottom and surface boundaries are kept at a constant temperature, whereas the side boundaries are thermally insulated.

Velocity boundary conditions are imposed on both sides of the model with a constant convergence velocity imposed on the topmost 120 km of the left boundary of the model (varied between 5 and 1 cm year^−1^; see Fig. 3), whereas the right boundary is kept fixed. The velocity boundary conditions between 120 and 660 km depth are defined so that the material influx and outflux through the lateral boundaries is in balance at any given time. In the constant viscosity lower mantle, the side boundaries are closed. At the base of the model, a free-slip condition is imposed and there is no material transport through this boundary. To restrict the effects of the slab’s interference with the bottom boundary of the model, the slab is transformed into a lower mantle material at 1200 km depth. The top of the model is a free surface.

## References

[1] B. Taylor, G. D. Karner, On the evolution of marginal basins. Rev. Geophys. 21, 1727–1741 (1983).

[2] G. S. Lister, M. A. Forster, T. J. Rawling, Episodicity during orogenesis. Geol. Soc. Lond. Spec. Pub. 184, 89–113 (2001).

[3] W. J. Collins, Hot orogens, tectonic switching, and creation of continental crust. Geology 30, 535–538 (2002).

[4] D. Jongsma, Heat flow in the Aegean Sea. Geophys. J. Int. 37, 337–346 (1974).

[5] S. C. Stiros, in *Terrestrial Heat Flow and the Lithosphere Structure* (Springer, 1991), chap. 19, pp. 395–416.

[6] I. Kassaras, V. Kapetanidis, A. Karakonstantis, P. Papadimitriou, Deep structure of the Hellenic lithosphere from teleseismic Rayleigh-wave tomography. Geophys. J. Int. 221, 205–230 (2020).

[7] J.-P. Brun, C. Faccenna, Exhumation of high-pressure rocks driven by slab rollback. Earth Planet. Sci. Lett. 272, 1–7 (2008).

[8] L. Jolivet, J.-P. Brun, Cenozoic geodynamic evolution of the Aegean. Int. J. Earth Sci. 99, 109–138 (2008).

[9] L. Jolivet, C. Faccenna, B. Huet, L. Labrousse, L. Le Pourhiet, O. Lacombe, E. Lecomte, E. Burov, Y. Denèle, J.-P. Brun, M. Philippon, A. Paul, G. Salaün, H. Karabulut, C. Piromallo, P. Monié, F. Gueydan, A. I. Okay, R. Oberhänsli, A. Pourteau, R. Augier, L. Gadenne, O. Driussi, Aegean tectonics: Strain localisation, slab tearing and trench retreat. Tectonophysics 597-598, 1–33 (2013).

[10] C. Tirel, J.-P. Brun, E. Burov, Dynamics and structural development of metamorphic core complexes. J. Geophys. Res. 113, B04403 (2008).

[11] L. Jolivet, Subduction tectonics and exhumation of high-pressure metamorphic rocks in the Mediterranean orogens. Am. J. Sci. 303, 353–409 (2003).

[12] D. J. J. van Hinsbergen, E. Hafkenscheid, W. Spakman, J. E. Meulenkamp, R. Wortel, Nappe stacking resulting from subduction of oceanic and continental lithosphere below Greece. Geology 33, 325–328 (2005).

[13] I. M. Artemieva, A. Shulgin, Geodynamics of Anatolia: Lithosphere thermal structure and thickness. Tectonics 38, 4465–4487 (2019).

[14] D. McKenzie, The structure of the lithosphere and upper mantle beneath the Eastern Mediterranean and Middle East. Mediterranean Geosci. Rev. 2, 311–326 (2020).

[15] D. Gürer, D. J. J. van Hinsbergen, Diachronous demise of the Neotethys Ocean as a driver for non-cylindrical orogenesis in Anatolia. Tectonophysics 760, 95–106 (2019).

[16] A. Menant, L. Jolivet, B. Vrielynck, Kinematic reconstructions and magmatic evolution illuminating crustal and mantle dynamics of the eastern Mediterranean region since the late Cretaceous. Tectonophysics 675, 103–140 (2016).

[17] J. F. Dewey, A. M. C. Şengör, Aegean and surrounding regions: Complex multiplate and continuum tectonics in a convergent zone. Geological Soc. Am. Bull. 90, 84–92 (1979).

[18] A. M. C. Şengör, Y. Yilmaz, Tethyan evolution of Turkey: A plate tectonic approach. Tectonophysics 75, 181–241 (1981).

[19] Y. Dilek, E. Sandvol, Seismic structure, crustal architecture and tectonic evolution of the Anatolian-African Plate Boundary and the Cenozoic Orogenic Belts in the Eastern Mediterranean Region. Geologic. Soc. Lond. Spec. Publ. 327, 127–160 (2009).

[20] D. J. J. van Hinsbergen, D. Gürer, A. Koç, N. Lom, Shortening and extrusion in the East Anatolian Plateau: How was Neogene Arabia-Eurasia convergence tectonically accommodated? Earth Planet. Sci. Lett. 641, 118827 (2024).

[21] D. J. J. van Hinsbergen, M. Maffione, A. Plunder, N. Kaymakcı, M. Ganerød, B. W. H. Hendriks, F. Corfu, D. Gürer, G. I. N. O. de Gelder, K. Peters, P. J. McPhee, F. M. Brouwer, E. L. Advokaat, R. L. M. Vissers, Tectonic evolution and paleogeography of the Kırşehir Block and the Central Anatolian Ophiolites, Turkey. Tectonics 35, 983–1014 (2016).

[22] D. J. J. van Hinsbergen, T. H. Torsvik, S. M. Schmid, L. C. Maţenco, M. Maffione, R. L. M. Vissers, D. Gürer, W. Spakman, Orogenic architecture of the Mediterranean region and kinematic reconstruction of its tectonic evolution since the Triassic. Gondw. Res. 81, 79–229 (2020).

[23] H. Gabrielse, C. Yorath, DNAG# 4. The Cordilleran Orogen in Canada. Geosci. Can. 16, 67–83 (1989).

[24] H. Gabrielse, C. J. Yorath, *Geology of the Cordilleran Orogen in Canada* (Geological Society of America, 1991).

[25] R. M. Clowes, C. A. Zelt, J. R. Amor, R. M. Ellis, Lithospheric structure in the southern Canadian Cordillera from a network of seismic refraction lines. Can. J. Earth Sci. 32, 1485–1513 (1995).

[26] W. R. Dickinson, Evolution of the North American Cordillera. Annu. Rev. Earth Planet. Sci. 32, 13–45 (2004).

[27] C. A. Currie, R. D. Hyndman, The thermal structure of subduction zone back arcs. J. Geophys. Res. 111, B08404 (2006).

[28] R. D. Hyndman, P. Flück, S. Mazzotti, T. J. Lewis, J. Ristau, L. Leonard, Current tectonics of the northern Canadian Cordillera. Can. J. Earth Sci. 42, 1117–1136 (2005).

[29] A. J. Wickens, The upper mantle of southern British Columbia. Can. J. Earth Sci. 14, 1100–1115 (1977).

[30] H. Yuan, S. French, P. Cupillard, B. Romanowicz, Lithospheric expression of geological units in central and eastern North America from full waveform tomography. Earth Planet. Sci. Lett. 402, 176–186 (2014).

[31] H. Bijwaard, W. Spakman, E. R. Engdahl, Closing the gap between regional and global travel time tomography. J. Geophys. Res. Solid Earth 103, 30055–30078 (1998).

[32] B. Endrun, S. Lebedev, T. Meier, C. Tirel, W. Friederich, Complex layered deformation within the Aegean crust and mantle revealed by seismic anisotropy. Nat. Geosci. 4, 203–207 (2011).

[33] B. Endrun, T. Meier, S. Lebedev, M. Bohnhoff, G. Stavrakakis, H.-P. Harjes, *S* velocity structure and radial anisotropy in the Aegean region from surface wave dispersion. Geophys. J. Int. 174, 593–616 (2008).

[34] E. E. Davis, T. J. Lewis, Heat flow in a back-arc environment: Intermontane and Omineca Crystalline belts, southern Canadian Cordillera. Can. J. Earth Sci. 21, 715–726 (1984).

[35] R. D. Hyndman, C. A. Currie, S. P. Mazzotti, Subduction zone backarcs, mobile belts, and orogenic heat. GSA Today 15, 4 (2005).

[36] M. Springer, A. Forster, Heat-flow density across the Central Andean subduction zone. Tectonophysics 291, 123–139 (1998).

[37] V. Cermak, L. Rybach, Eds., *Terrestrial Heat Flow in Europe* (Springer, 1979), p. 328.

[38] P. J. Coney, D. L. Jones, J. W. H. Monger, Cordilleran suspect terranes. Nature 288, 329–333 (1980).

[39] J. Jackson, Active tectonics of the Aegean region. Annu. Rev. Earth Planet. Sci. 22, 239–271 (1994).

[40] C. A. Currie, R. S. Huismans, C. Beaumont, Thinning of continental backarc lithosphere by flow-induced gravitational instability. Earth Planet. Sci. Lett. 269, 436–447 (2008).

[41] C. A. Currie, R. D. Hyndman, Reply to comment by W. P. Schellart on “The thermal structure of subduction zone back arcs”. J. Geophys. Res. 112, B11408 (2007).

[42] Z. Erdős, R. S. Huismans, C. Faccenna, Wide versus narrow back-arc rifting: Control of subduction velocity and convective back-arc thinning. Tectonics 41, e2021TC007086 (2022).

[43] S. G. Wolf, R. S. Huismans, Mountain building or backarc extension in ocean-continent subduction systems: A function of backarc lithospheric strength and absolute plate velocities. J. Geophys. Res. Solid Earth 124, 7461–7482 (2019).

[44] P. Bird, Continental delamination and the Colorado Plateau. J. Geophys. Res. Solid Earth 84, 7561–7571 (1979).

[45] O. H. Göğüş, K. Ueda, Peeling back the lithosphere: Controlling parameters, surface expressions and the future directions in delamination modeling. J. Geodyn. 117, 21–40 (2018).

[46] R. W. Kay, S. Mahlburg-Kay, Creation and destruction of lower continental crust. Geol. Rundsch. 80, 259–278 (1991).

[47] B. R. Hacker, P. B. Kelemen, M. D. Behn, Differentiation of the continental crust by relamination. Earth Planet. Sci. Lett. 307, 501–516 (2011).

[48] W. P. Schellart, Comment on “The thermal structure of subduction zone back arcs” by Claire A. Currie and Roy D. Hyndman. J. Geophys. Res. 112, B11407 (2007).

[49] C. A. Currie, M. N. Ducea, P. G. DeCelles, C. Beaumont, in *Geodynamics of a Cordilleran Orogenic System: The Central Andes of Argentina and Northern Chile* (Geological Society of America, 2015).

[50] P. G. DeCelles, M. N. Ducea, P. Kapp, G. Zandt, Cyclicity in Cordilleran orogenic systems. Nat. Geosci. 2, 251–257 (2009).

[51] C. Tirel, J. P. Brun, E. Burov, M. J. R. Wortel, S. Lebedev, A plate tectonics oddity: Caterpillar-walk exhumation of subducted continental crust. Geology 41, 555–558 (2013).

[52] J. L. Tetreault, S. J. H. Buiter, Geodynamic models of terrane accretion: Testing the fate of island arcs, oceanic plateaus, and continental fragments in subduction zones. J. Geophys. Res. Solid Earth 117, B08403 (2012).

[53] J. L. Tetreault, S. J. H. Buiter, Future accreted terranes: A compilation of island arcs, oceanic plateaus, submarine ridges, seamounts, and continental fragments. Solid Earth 5, 1243–1275 (2014).

[54] R. W. Bialas, F. Funiciello, C. Faccenna, Subduction and exhumation of continental crust: Insights from laboratory models. Geophys. J. Int. 184, 43–64 (2011).

[55] C. Thieulot, FANTOM: Two- and three-dimensional numerical modelling of creeping flows for the solution of geological problems. Phys. Earth Planet. Inter. 188, 47–68 (2011).

[56] Z. Erdős, R. S. Huismans, P. van der Beek, C. Thieulot, Extensional inheritance and surface processes as controlling factors of mountain belt structure. J. Geophys. Res. Solid Earth 119, 9042–9061 (2014).

[57] A. L. Perchuk, T. V. Gerya, V. S. Zakharov, W. L. Griffin, Building cratonic keels in Precambrian plate tectonics. Nature 586, 395–401 (2020). 33057224 10.1038/s41586-020-2806-7

[58] W. P. Schellart, Influence of the subducting plate velocity on the geometry of the slab and migration of the subduction hinge. Earth Planet. Sci. Lett. 231, 197–219 (2005).

[59] C. Faccenna, T. W. Becker, L. Auer, A. Billi, L. Boschi, J. P. Brun, F. A. Capitanio, F. Funiciello, F. Horvath, L. Jolivet, C. Piromallo, L. Royden, F. Rossetti, E. Serpelloni, Mantle dynamics in the Mediterranean. Rev. Geophys. 52, 283–332 (2014).

[60] C. Faccenna, M. Mattei, R. Funiciello, L. Jolivet, Styles of back-arc extension in the Central Mediterranean. Terra Nova 9, 126–130 (1997).

[61] L. Royden, C. Faccenna, Subduction orogeny and the Late Cenozoic evolution of the Mediterranean Arcs. Annu. Rev. Earth Planet. Sci. 46, 261–289 (2018).

[62] S. M. Schmid, D. Bernoulli, B. Fügenschuh, L. Matenco, S. Schefer, R. Schuster, M. Tischler, K. Ustaszewski, The Alpine-Carpathian-Dinaridic orogenic system: Correlation and evolution of tectonic units. Swiss J. Geosci. 101, 139–183 (2008).

[63] S. M. Schmid, B. Fügenschuh, A. Kounov, L. Maţenco, P. Nievergelt, R. Oberhänsli, J. Pleuger, S. Schefer, R. Schuster, B. Tomljenović, K. Ustaszewski, D. J. J. van Hinsbergen, Tectonic units of the Alpine collision zone between Eastern Alps and western Turkey. Gondw. Res. 78, 308–374 (2020).

[64] L. Jolivet, C. Faccenna, Mediterranean extension and the Africa-Eurasia collision. Tectonics 19, 1095–1106 (2000).

[65] S. Agostini, C. Doglioni, F. Innocenti, P. Manetti, S. Tonarini, On the geodynamics of the Aegean rift. Tectonophysics 488, 7–21 (2010).

[66] X. Le Pichon, J. Angelier, M. F. Osmaston, L. Stegena, The Aegean Sea [and Discussion]. Philos. Trans. R. Soc. London Ser. A Math. Phys. Eng. Sci. 300, 357–372 (1981).

[67] M. J. R. Wortel, S. D. B. Goes, W. Spakman, Structure and seismicity of the Aegean subduction zone. Terra Nova 2, 554–562 (1990).

[68] D. J. J. van Hinsbergen, S. M. Schmid, Map view restoration of Aegean-West Anatolian accretion and extension since the Eocene. Tectonics 31, TC5005 (2012).

[69] A. I. Al-Lazki, D. Seber, E. Sandvol, N. Turkelli, R. Mohamad, M. Barazangi, Tomographic Pn velocity and anisotropy structure beneath the Anatolian plateau (eastern Turkey) and the surrounding regions. Geophys. Res. Lett. 30, 8043 (2003).

[70] R. Gök, E. Sandvol, N. Türkelli, D. Seber, M. Barazangi, Sn attenuation in the Anatolian and Iranian plateau and surrounding regions. Geophys. Res. Lett. 30, 8042 (2003).

[71] S. Altunkaynak, Y. Dilek, in *Postcollisional Tectonics and Magmatism in the Mediterranean Region and Asia* (Geological Society of America, 2006), pp. 321–351.

[72] M. Colpron, J. L. Nelson, D. C. Murphy, Northern Cordilleran terranes and their interactions through time. GSA Today 17, 4 (2007).

[73] R. D. Hyndman, C. A. Currie, Why is the North America Cordillera high? Hot backarcs, thermal isostasy, and mountain belts. Geology 39, 783–786 (2011).

[74] G. Lister, M. Forster, Tectonic mode switches and the nature of orogenesis. Lithos 113, 274–291 (2009).

[75] A. L. Perchuk, O. G. Safonov, C. A. Smit, D. D. van Reenen, V. S. Zakharov, T. V. Gerya, Precambrian ultra-hot orogenic factory: Making and reworking of continental crust. Tectonophysics 746, 572–586 (2018).

[76] P. Chowdhury, S. Chakraborty, T. V. Gerya, P. A. Cawood, F. A. Capitanio, Peel-back controlled lithospheric convergence explains the secular transitions in Archean metamorphism and magmatism. Earth Planet. Sci. Lett. 538, 116224 (2020).

[77] A. M. C. Sengör, Mid-Mesozoic closure of Permo–Triassic Tethys and its implications. Nature 279, 590–593 (1979).

[78] A. Zanchi, S. Zanchetta, E. Garzanti, M. Balini, F. Berra, M. Mattei, G. Muttoni, The Cimmerian evolution of the Nakhlak–Anarak area, Central Iran, and its bearing for the reconstruction of the history of the Eurasian margin. Geol. Soc. Lond. Spec. Publ. 312, 261–286 (2009).

[79] J. F. Dewey, R. M. Shackleton, C. Chengfa, S. Yiyin, The tectonic evolution of the Tibetan Plateau. Philos. Trans. R. Soc. London Ser. A Math. Phys. Sci. 327, 379–413 (1988).

[80] A. Yin, T. M. Harrison, Geologic evolution of the Himalayan-Tibetan orogen. Annu. Rev. Earth Planet. Sci. 28, 211–280 (2000).

[81] P. Kapp, P. G. DeCelles, Mesozoic–Cenozoic geological evolution of the Himalayan-Tibetan orogen and working tectonic hypotheses. Am. J. Sci. 319, 159–254 (2019).

[82] W. P. Schellart, Subduction dynamics and overriding plate deformation. Earth Sci. Rev. 253, 104755 (2024).

[83] W. P. Schellart, J. Freeman, D. R. Stegman, L. Moresi, D. May, Evolution and diversity of subduction zones controlled by slab width. Nature 446, 308–311 (2007). 17361181 10.1038/nature05615

[84] L. Moresi, P. G. Betts, M. S. Miller, R. A. Cayley, Dynamics of continental accretion. Nature 508, 245–248 (2014). 24670638 10.1038/nature13033

[85] K. Vogt, T. V. Gerya, From oceanic plateaus to allochthonous terranes: Numerical modelling. Gondw. Res. 25, 494–508 (2014).

[86] Z. Yan, L. Chen, A. V. Zuza, Q. Meng, Successive accretions of future allochthonous terranes and multiple subduction zone jumps: Implications for Tethyan evolution. Geol. Soc. Am. Bull. 136, 3230–3242 (2024).

[87] Z. Erdős, R. S. Huismans, C. Faccenna, S. G. Wolf, The role of subduction interface and upper plate strength on back-arc extension: Application to mediterranean back-arc basins. Tectonics 40, e2021TC006795 (2021).

[88] R. Huismans, C. Beaumont, Depth-dependent extension, two-stage breakup and cratonic underplating at rifted margins. Nature 473, 74–78 (2011). 21544144 10.1038/nature09988

[89] G. Hirth, D. L. Kohlstedt, Water in the oceanic upper mantle: Implications for rheology, melt extraction and the evolution of the lithosphere. Earth Planet. Sci. Lett. 144, 93–108 (1996).

[90] S. Karato, P. Wu, Rheology of the upper mantle: A synthesis. Science 260, 771–778 (1993). 17746109 10.1126/science.260.5109.771

[91] R. H. Sibson, Conditions for fault-valve behaviour. Geol. Soc. Spec. Publ. 54, 15–28 (1990).

[92] B. Bos, C. J. Spiers, Frictional-viscous flow of phyllosilicate-bearing fault rock: Microphysical model and implications for crustal strength profiles. J. Geophys. Res. Solid Earth 107, ECV 1-1–ECV 1-13 (2002).

[93] R. Agrusta, S. Goes, J. van Hunen, Subducting-slab transition-zone interaction: Stagnation, penetration and mode switches. Earth Planet. Sci. Lett. 467, 10–23 (2017).

[94] J. P. Butler, C. Beaumont, R. A. Jamieson, Paradigm lost: Buoyancy thwarted by the strength of the Western Gneiss Region (ultra)high-pressure terrane, Norway. Lithosphere 7, 379–407 (2015).

[95] N. Tosi, D. A. Yuen, N. de Koker, R. M. Wentzcovitch, Mantle dynamics with pressure- and temperature-dependent thermal expansivity and conductivity. Phys. Earth Planet. Inter. 217, 48–58 (2013).

[96] S. J. Mackwell, M. E. Zimmerman, D. L. Kohlstedt, High-temperature deformation of dry diabase with application to tectonics on Venus. J. Geophys. Res. Solid Earth 103, 975–984 (1998).

[97] G. C. Gleason, J. Tullis, A flow law for dislocation creep of quartz aggregates determined with the molten-salt cell. Tectonophysics 247, 1–23 (1995).

[98] B. R. Hacker, in *Subduction Top to Bottom*, G. E. Bebout, D. W. Scholl, S. H. Kirby, J. P. Platt, Eds. (Wiley, 1996), pp. 337–346.

[99] M. I. Billen, Slab dynamics in the transition zone. Phys. Earth Planet. Inter. 183, 296–308 (2010).

[100] S. Goes, R. Agrusta, J. van Hunen, F. Garel, Subduction-transition zone interaction: A review. Geosphere 13, 644–664 (2017).

[101] J. X. Mitrovica, A. M. Forte, A new inference of mantle viscosity based upon joint inversion of convection and glacial isostatic adjustment data. Earth Planet. Sci. Lett. 225, 177–189 (2004).

[102] C. Amante, B. W. Eakins, ETOPO1 arc-minute global relief model: Procedures, data sources and analysis (NOAA Technical Memorandum NESDIS NGDC-24, NOAA, 2009).

[103] C. Kreemer, W. E. Holt, A. J. Haines, An integrated global model of present-day plate motions and plate boundary deformation. Geophys. J. Int. 154, 8–34 (2003).

[104] P. Bird, An updated digital model of plate boundaries. Geochem. Geophys. Geosyst. 4, 1027 (2003).

[105] R. D. Hyndman, T. J. Lewis, Geophysical consequences of the Cordillera–Craton thermal transition in southwestern Canada. Tectonophysics 306, 397–422 (1999).

