# Supplementary material for: Terrane accretion explains thin and hot ocean-continent back-arcs
Source: Sci Adv. 2025 Apr 25;11(17):eadq8444. doi: 10.1126/sciadv.adq8444 (PMC12024691; doi:10.1126/sciadv.adq8444)
Supplement: Supplementary file 1 — Fig. S1 Table S1 Legends for movies S1 to S15 [file sciadv.adq8444_sm.pdf]

Science Advances
AAAS

Supplementary Materials for

Terrane accretion explains thin and hot ocean-continent back-arcs

Zoltán Erdős *et al.*

Corresponding author: Zoltán Erdős, erdoes@gfz.de; Ritske S. Huismans, ritske.huismans@uib.no

Sci. Adv. **11**, eadq8444 (2025)
DOI: 10.1126/sciadv.adq8444

The PDF file includes:

Fig. S1
Table S1
Legends for movies S1 to S15

Other Supplementary Material for this manuscript includes the following:

Movies S1 to S15

Supplementary Text

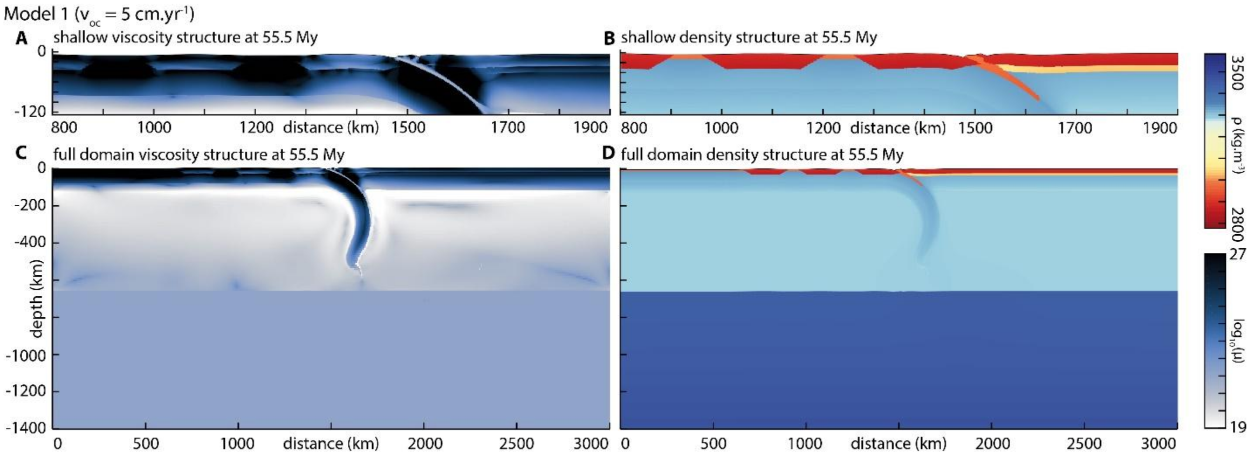

Fig. S1: Density and viscosity structure of the experiments through representative snapshots of Model 1. All snapshots taken at 55.5 myr (at the initiation of the first microcontinent collision). **A)** Shallow viscosity structure; **B)** Shallow density structure; **C)** Full domain viscosity structure; **D)** Full domain density structure.

Experiment ID	Convergence velocity ($cmyr^{-1}$)	Mantle depletion (kgm^{-3})
M1	2	15
M2	5	15
SM1	5	40
SM2	5	30
SM3	4	40
SM4	4	30
SM5	4	15
SM6	3	40
SM7	3	30
SM8	3	15
SM9	2	40
SM10	2	30
SM11	1	40
SM12	1	30
SM13	1	15

Supplementary table S1: List of all experiments and the varied parameters.

Movie S1: Evolution of Model 1 through time. The top panel displays the evolution of the central, shallow part of the model domain. The colors represent the material distribution (see Fig. 5). The bottom panel displays the evolution of the central domain at an upper-mantle scale. The colors represent the viscosity field.

Movie S2: Evolution of Model 2 through time. The top panel displays the evolution of the central, shallow part of the model domain. The colors represent the material distribution (see Fig. 5). The bottom panel displays the evolution of the central domain at an upper-mantle scale. The colors represent the viscosity field.

Movie S3: Evolution of Supplementary model SM1 through time. The top panel displays the evolution of the central, shallow part of the model domain. The colors represent the material distribution (see Fig. 5). The bottom panel displays the evolution of the central domain at an upper-mantle scale. The colors represent the viscosity field.

Movie S4: Evolution of Supplementary model SM2 through time. The top panel displays the evolution of the central, shallow part of the model domain. The colors represent the material distribution (see Fig. 5). The bottom panel displays the evolution of the central domain at an upper-mantle scale. The colors represent the viscosity field.

Movie S5: Evolution of Supplementary model SM3 through time. The top panel displays the evolution of the central, shallow part of the model domain. The colors represent the material distribution (see Fig. 5). The bottom panel displays the evolution of the central domain at an upper-mantle scale. The colors represent the viscosity field.

Movie S6: Evolution of Supplementary model SM4 through time. The top panel displays the evolution of the central, shallow part of the model domain. The colors represent the material distribution (see Fig. 5). The bottom panel displays the evolution of the central domain at an upper-mantle scale. The colors represent the viscosity field.

Movie S7: Evolution of Supplementary model SM5 through time. The top panel displays the evolution of the central, shallow part of the model domain. The colors represent the material distribution (see Fig. 5). The bottom panel displays the evolution of the central domain at an upper-mantle scale. The colors represent the viscosity field.

Movie S8: Evolution of Supplementary model SM6 through time. The top panel displays the evolution of the central, shallow part of the model domain. The colors represent the material distribution (see Fig. 5). The bottom panel displays the evolution of the central domain at an upper-mantle scale. The colors represent the viscosity field.

Movie S9: Evolution of Supplementary model SM7 through time. The top panel displays the evolution of the central, shallow part of the model domain. The colors represent the material distribution (see Fig. 5). The bottom panel displays the evolution of the central domain at an upper-mantle scale. The colors represent the viscosity field.

Movie S10: Evolution of Supplementary model SM8 through time. The top panel displays the evolution of the central, shallow part of the model domain. The colors represent the material distribution (see Fig. 5). The bottom panel displays the evolution of the central domain at an upper-mantle scale. The colors represent the viscosity field.

Movie S11: Evolution of Supplementary model SM9 through time. The top panel displays the evolution of the central, shallow part of the model domain. The colors represent the material distribution (see Fig. 5). The bottom panel displays the evolution of the central domain at an upper-mantle scale. The colors represent the viscosity field.

Movie S12: Evolution of Supplementary model SM10 through time. The top panel displays the evolution of the central, shallow part of the model domain. The colors represent the material distribution (see Fig. 5). The bottom panel displays the evolution of the central domain at an upper-mantle scale. The colors represent the viscosity field.

Movie S13: Evolution of Supplementary model SM11 through time. The top panel displays the evolution of the central, shallow part of the model domain. The colors represent the material distribution (see Fig. 5). The bottom panel displays the evolution of the central domain at an upper-mantle scale. The colors represent the viscosity field.

Movie S14: Evolution of Supplementary model SM12 through time. The top panel displays the evolution of the central, shallow part of the model domain. The colors represent the material distribution (see Fig. 5). The bottom panel displays the evolution of the central domain at an upper-mantle scale. The colors represent the viscosity field.

Movie S15: Evolution of Supplementary model SM13 through time. The top panel displays the evolution of the central, shallow part of the model domain. The colors represent the material distribution (see Fig. 5). The bottom panel displays the evolution of the central domain at an upper-mantle scale. The colors represent the viscosity field.
